# Increasing Incidence of Colorectal Cancer in Adolescents and Young Adults Aged 15–39 Years in Western Australia 1982–2007: Examination of Colonoscopy History

**DOI:** 10.3389/fpubh.2017.00179

**Published:** 2017-07-24

**Authors:** Lakkhina Troeung, Nita Sodhi-Berry, Angelita Martini, Eva Malacova, Hooi Ee, Peter O’Leary, Iris Lansdorp-Vogelaar, David B. Preen

**Affiliations:** ^1^Centre for Health Services Research, School of Population Health, The University of Western Australia, Perth, WA, Australia; ^2^Occupational Respiratory Epidemiology, School of Population Health, The University of Western Australia, Perth, WA, Australia; ^3^Department of Health, Safety and Environment, School of Public Health, Curtin University, Perth, WA, Australia; ^4^Department of Gastroenterology, Sir Charles Gairdner Hospital, Queen Elizabeth II Medical Centre, Nedlands, WA, Australia; ^5^Health Policy and Management, Faculty of Health Sciences, School of Public Health, Curtin University, Perth, WA, Australia; ^6^School of Women’s and Infants’ Health, The University of Western Australia, Perth, WA, Australia; ^7^Department of Public Health, Erasmus MC University Medical Centre, Rotterdam, Netherlands

**Keywords:** colorectal cancer screening, young adults, colonoscopy, colorectal cancer, incidence trends

## Abstract

**Aims:**

To examine trends in colorectal cancer (CRC) incidence and colonoscopy history in adolescents and young adults (AYAs) aged 15–39 years in Western Australia (WA) from 1982 to 2007.

**Design:**

Descriptive cohort study using population-based linked hospital and cancer registry data.

**Method:**

Five-year age-standardized and age-specific incidence rates of CRC were calculated for all AYAs and by sex. Temporal trends in CRC incidence were investigated using Joinpoint regression analysis. The annual percentage change (APC) in CRC incidence was calculated to identify significant time trends. Colonoscopy history relative to incident CRC diagnosis was examined and age and tumor grade at diagnosis compared for AYAs with and without pre-diagnosis colonoscopy. CRC-related mortality within 5 and 10 years of incident diagnosis were compared for AYAs with and without pre-diagnosis colonoscopy using mortality rate ratios (MRRs) derived from negative binomial regression.

**Results:**

Age-standardized CRC incidence among AYAs significantly increased in WA between 1982 and 2007, APC = 3.0 (95% CI 0.7–5.5). Pre-diagnosis colonoscopy was uncommon among AYAs (6.0%, 33/483) and 71% of AYAs were diagnosed after index (first ever) colonoscopy. AYAs with pre-diagnosis colonoscopy were older at CRC diagnosis (mean 36.7 ± 0.7 years) compared to those with no prior colonoscopy (32.6 ± 0.2 years), *p* < 0.001. At CRC diagnosis, a significantly greater proportion of AYAs with pre-diagnosis colonoscopy had well-differentiated tumors (21.2%) compared to those without (5.6%), *p* = 0.001. CRC-related mortality was significantly lower for AYAs with pre-diagnosis colonoscopy compared to those without, for both 5-year [MRR = 0.44 (95% CI 0.27–0.75), *p* = 0.045] and 10-year morality [MRR = 0.43 (95% CI 0.24–0.83), *p* = 0.043].

**Conclusion:**

CRC incidence among AYAs in WA has significantly increased over the 25-year study period. Pre-diagnosis colonoscopy is associated with lower tumor grade at CRC diagnosis as well as significant reduction in both 5- and 10-year CRC-related mortality rates. These findings warrant further research into the balance in benefits and harms of targeted screening for AYA at highest risk.

## Introduction

Australia and New Zealand have the highest rates of colorectal cancer (CRC) internationally (1). The average age at incident CRC diagnosis is 70 years with sharp increases in incidence from 50 years of age (2). Accordingly, current Australian guidelines recommend biennial CRC screening through fecal occult blood tests commencing from 50 years of age for all asymptomatic average-risk persons (3). In the United States (US), CRC incidence and mortality in persons over 50 years have declined over the past decade owing in part to screening initiatives (4). In particular, increased uptake of screening colonoscopy is suggested to be the main driver of declining CRC rates in this age group (5), with early detection and removal of premalignant lesions yielding significant reductions in CRC morbidity and mortality (6–10).

In direct contrast to trends in those over 50 years of age, an increasing incidence of CRC among adolescents and young adults (AYAs) has been reported internationally (11–15) as well as in Australia (16, 17) over the past two decades. A recent report showed that from 1990 to 2010, CRC incidence increased by between 85 and 100% in Australians aged 20–29 years and by 35% in those aged 30–39 years (17). The mechanisms underlying the rising incidence of CRC among AYAs are currently not well understood (15, 18); however, this increasing trend is a population health concern (18). Given the observed benefits of screening colonoscopy in the older population (5), questions have been raised in relation to current CRC screening practices in younger populations and whether average-risk CRC screening should be initiated at an earlier age (18–20). However, there is currently a lack of empirical data on the impact of screening in age groups <50 years to inform decision-making.

We examined trends in CRC incidence and colonoscopy history among AYAs aged 15–39 years in Western Australia (WA) from 1982 to 2007, before implementation of the National Bowel Cancer Screening Program (NBCSP), using whole-population linked hospital and Cancer Registry data. While the AYA age group is currently exempt from the NBCSP framework, it is possible that raised awareness of bowel cancer through the NBCSP may have impacted screening behaviors in the younger population. We therefore selected 2007 as our endpoint to examine pre-NBCSP colonoscopy history in AYAs. Specifically, we sought to (1) examine temporal trends in age-standardized and age-specific CRC incidence rates, (2) examine colonoscopy history in AYAs, and (3) compare age at diagnosis, tumor grade, and 10-year CRC-related mortality for AYAs with and without a record of pre-diagnosis colonoscopy.

## Materials and Methods

### Data Sources

Data were obtained on all persons aged 15–39 years with an incident diagnosis of malignant neoplasm in WA between 1st January 1982 and 31st December 2007, as registered with the WA Cancer Registry (WACR). The age range of 15–39 years for AYA classification is based on that used previously (16, 21). Since 1981, notifications of all malignancies within 6 months of diagnosis have been a statutory requirement in WA, with 86% of cases confirmed histologically (22). Extracted WACR records included information on sociodemographic (age, sex, Indigenous status, and area of residence) and tumor characteristics (diagnosis date, tumor site, morphology, behavior, and grade). Hospital records from 1982 to 2007 for the cohort were obtained through probabilistic matching of WACR records to the WA Hospital Morbidity Data System (HMDS) through the WA Data Linkage System (23). The HMDS is a statutory data collection which captures data for all public and private hospitalizations in WA. All colonoscopies in WA are hospital-based procedures and thus captured in the HMDS. Death records for cohort were also obtained through linkage with the WA Mortality Registry (1982–2007).

### Trends in CRC Incidence

Incident primary cases of CRC were ascertained from WACR records using the International Classification of Diseases (ICD) version 9 with Clinical Modifications (ICD-9-CM) codes (153-154) and ICD version 10 with Australian Modification (ICD-10-AM) codes (C18-C21). Incidence rates were calculated by including only the first-ever primary CRC diagnosis for each person (i.e., subsequent CRC diagnoses, even if at different sites, were not counted). Persons registered in the WACR with another type of malignancy prior to CRC diagnosis were included, with date of first-ever CRC used for the analysis.

Five-year age-specific and age-standardized incidence rates of CRC were calculated for all AYAs and by sex using the number of incident CRC cases for each age group in each period as the numerator and the corresponding WA population for each age group in each period as the denominator. Denominators were obtained from population estimates provided by the Australian Bureau of Statistics (24). Age-standardized rates were adjusted by direct standardization against the 5-year age distribution of the Australian population in the 2001 Census.

Temporal trends in CRC incidence over the study period were investigated using Joinpoint regression analyses (25). Joinpoint analysis uses an algorithm to define segments where statistically significant changes in temporal trends occur. The annual percentage change (APC) in each Joinpoint segment represents the rate of change in cancer incidence per year in a given time period and is calculated using generalized linear models assuming a Poisson distribution (26). Changes in rates include shifts in magnitude or direction where a positive APC indicates an increase in cancer incidence for a given segment while a negative APC indicates decreasing incidence. Joinpoint regression analyses were performed using the Joinpoint Regression Program 4.3.1 from the US National Cancer Institute (25).

### Colonoscopy History

Hospital admissions for colonoscopy were ascertained from any of the 11 procedure fields in HMDS records using ICD-9-CM codes (45.21, 45.22, 45.23, 45.24, 45.25, 45.42, 48.24) for admissions between January 1982 and June 1999 and ICD-10-AM codes (32090-00, 56549-01, 32090-02, 32090-01, 90308-00, 90959-00, 90315-00, 32093-00, 32023-00, 32023-03, 32093-00, 32023-02, 32023-01, 32023-05, 32023-04, 32023-01, 92097-02, 32090-00, 32084-00, 32084-02, 32084-01, 90308-00, 90959-00, 90315-00, 32087-00, 30375-23, 56549-01, 32075-00, 32075-01, 32078-00, 32081-00) for hospitalizations from July 1999 onward. We incorporated a 1-year clearance period which excluded 18 AYAs diagnosed with CRC in 1982. A further 16 cases were excluded as they had no hospital records prior to or during the period of cancer diagnosis from which colonoscopy history could be ascertained.

To describe the cohort’s colonoscopy history, we divided all colonoscopies into three categories based on the timing of colonoscopy relative to incident CRC diagnosis. “Pre-diagnosis” colonoscopies were defined as any recorded colonoscopy greater than 6 months preceding the date of incident CRC diagnosis as registered with the WACR. “Diagnostic” colonoscopies were defined as any colonoscopies performed which resulted in a diagnosis of CRC within 6 months. “Post-diagnosis” colonoscopies were defined as any colonoscopy admission occurring after date of incident CRC diagnosis. Due to the limitations of administrative data and ICD coding standards, we were unable to determine whether pre-diagnosis colonoscopies were screening/surveillance (i.e., asymptomatic) or diagnostic colonoscopies (i.e., symptomatic colonoscopy).

Age and tumor grade at incident CRC diagnosis was compared between AYAs with and without a record of pre-diagnosis colonoscopy using *t*-tests and chi-square tests. Tumor grade was examined as data on cancer stage is not documented in the WACR.

### CRC Mortality

Deaths within 5 and 10 years of incident CRC diagnosis were identified using WA Death Registry records. CRC-related deaths were ascertained from the underlying cause of death field in death records using the following codes: ICD-9-CM 153-154 and ICD-10-AM C18-C21. CRC-related mortality rate ratios (MRRs) were compared for AYAs with and without pre-diagnosis colonoscopy using negative binomial regression to account for overdispersion of death data in Stata 14.0. Analyses were adjusted for sex and age at incident CRC diagnosis and Charlson comorbidity index. We restricted our analysis to only individuals who had 5 (i.e., diagnosed 1982–2002; *n* = 251) and 10 years (i.e., diagnosed 1982–1997; *n* = 234) of follow-up time, respectively. Differential person-years of risk for each person were accounted for by including time at risk as an offset variable in negative binomial models. Analysis of mortality rates was selected over survival rates to minimize the effect of lead-time bias commonly observed in cancer screening studies.

### Ethics Statement

Ethics approval for this study was obtained from the University of Western Australia Research Ethics Committees (reference number: RA/4/1/2228).

## Results

A total of 517 incident cases of CRC among AYAs aged 15–39 years were registered with the WACR between 1982 and 2007. There were 256 females (49.6%) and 261 males (50.4%). Mean age at incident CRC diagnosis was 33.7 ± 5.3 years (range 15.2–39.9 years). CRC accounted for 4.2% of all cancers diagnosed in AYAs between 1982 and 2007 in WA.

### CRC Incidence and Trends

**Figure 1:**
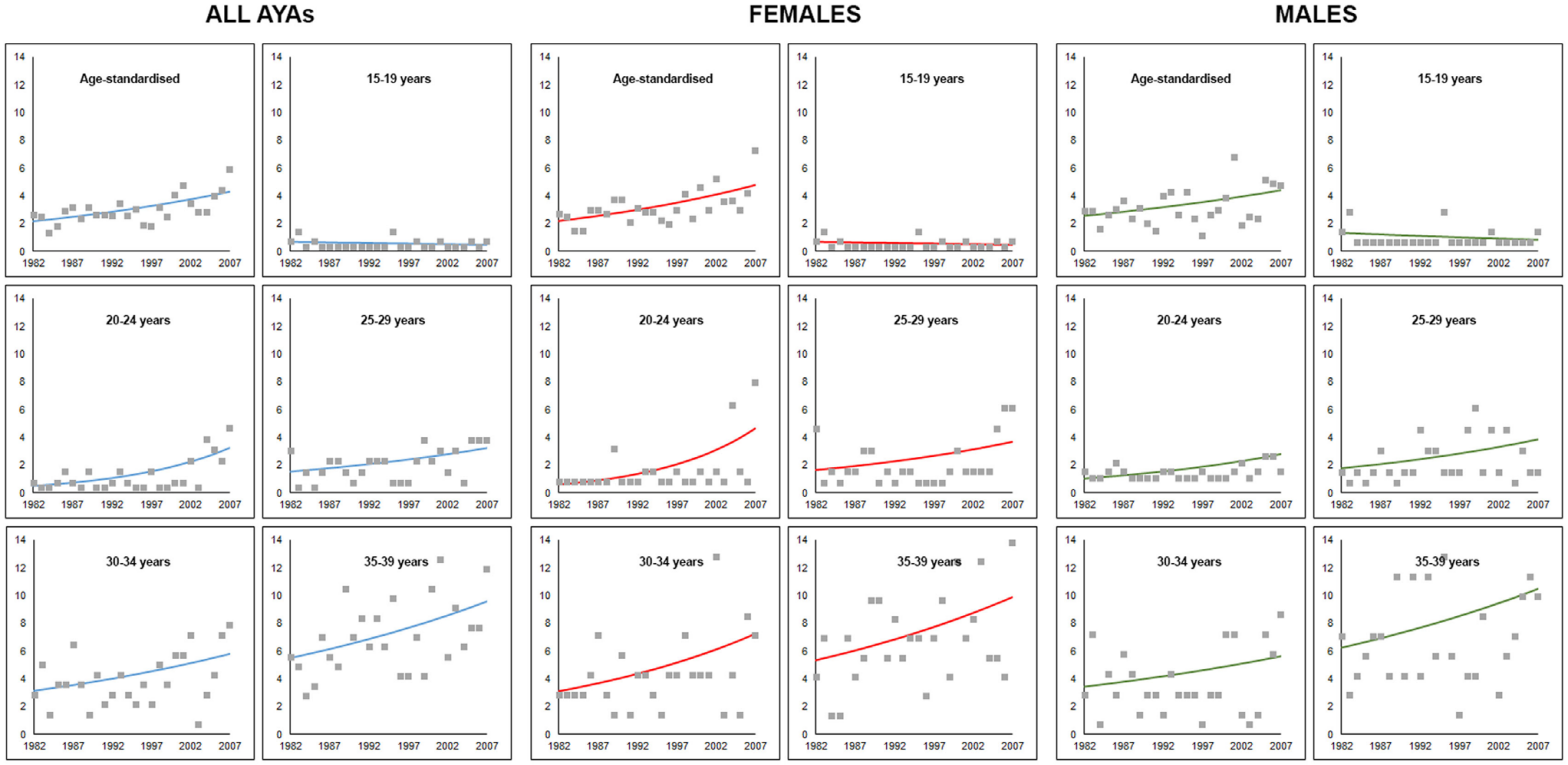
Trends in age-specific and age-adjusted incidence of colorectal cancer for adolescents and young adults aged 15–39 years in Western Australia, 1982–2007. Markers represent observed incidence rates and solid lines represent the Joinpoint regression model trend line.

Five-year age-standardized and age-specific incidence rates for CRC in AYAs are presented in Table 1 alongside Joinpoint regression results using annual incidence data. An increasing trend in age-standardized incidence rates for CRC in AYAs was observed over the study period (Figure 1). The overall age-standardized incidence of CRC significantly increased from 2.1 to 4.8 per 100,000 AYA population between 1982 and 2007, APC = 3.0 (95% CI 0.7–5.5), *p* = 0.024 (Table 1). The age-standardized incidence of CRC among female AYAs also significantly increased over the study period, APC = 3.4 (95% CI 1.1–5.7), *p* = 0.014. While an increasing trend in CRC incidence was observed for male AYAs, this was not statistically significant, APC = 2.6 (95% CI −1.0 to 5.2), *p* = 0.06.

**Table 1:** Five-year age-specific and age-standardized and Joinpoint analysis of annual colorectal cancer incidence rates per 100,000 in adolescents and young adults aged 15–39 years in Western Australia during 1982–2007.

	1982–1984	1985–1989	1990–1994	1995–1999	2000–2004	2005–2007	APC (95% CI)	
							1982–2007^a^	*p*
**All persons**								
**Age-specific rates**								
15–19 years	0.7	0.1	0.1	0.4	0.1	0.5	2.4 (−10.5 to 17.2)	0.649
20–24 years	0.3	0.9	0.6	0.3	1.6	3.4	8.3 (−2.6 to 20.4)	0.106
25–29 years	1.5	1.5	1.8	1.7	2.1	3.8	3.6 (0.6–6.8)*	0.029
30–34 years	3.1	3.7	3.3	3.3	4.5	6.5	2.7 (0.1–5.4)*	0.050
35–39 years	4.4	6.3	7.3	5.9	8.8	9.1	2.4 (0.1–4.7)*	0.047
Age-standardized rate	2.1	2.6	2.7	2.4	3.5	4.8	3.0 (0.7–5.5)*	0.024
**Males**								
**Age-specific rates**								
15–19 years	1.4	0.1	0.1	0.6	0.3	0.5	−4.9 (−14.7 to 6.0)	0.269
20–24 years	0.5	1.2	0.6	0.3	1.2	3.6	6.6 (−2.2 to 16.1)	0.110
25–29 years	1.0	1.2	2.8	3.1	2.4	2.0	2.4 (−4.0 to 9.2)	0.373
30–34 years	3.4	3.8	2.9	2.3	3.5	7.2	2.7 (−2.4 to 8.0)	0.235
35–39 years	4.7	7.1	7.4	5.7	8.5	10.4	2.2 (−0.6 to 5.0)	0.105
Age-standardized rate	2.3	2.8	2.8	2.4	3.3	4.8	2.6 (−0.9 to 5.2)	0.061
**Females**								
**Age-specific rates**								
15–19 years	0.2	0.3	0.1	0.3	0.1	0.5	2.0 (−4.3 to 8.7)	0.426
20–24 years	0.3	0.6	0.6	0.3	1.9	3.2	10.1 (3.3–17.5)*	0.014
25–29 years	2.0	1.8	0.9	0.3	1.8	5.6	4.9 (1.8–14.3)*	0.050
30–34 years	2.9	3.7	3.7	4.3	5.4	5.7	2.8 (1.6–4.0)*	0.002
35–39 years	4.2	5.5	7.2	6.1	9.1	7.8	2.6 (0.1–5.3)*	0.050
Age-standardized rate	2.0	2.5	2.7	2.4	3.9	4.7	3.4 (1.1–5.7)*	0.014

*APC, annual percentage change*.

*APC is statistically significant at a 0.05 level.

*^a^The model with 0 Joinpoints (i.e., 1982–2007) was most optimal in all analyses*.

An upward trend in CRC incidence was observed in all age groups but the 15–19 years category, for both males and females (Figure 1). However, none of the trends were statistically significant for males. For female AYAs, significant increases in CRC incidence were observed across all age groups except in the 15- to 19-year group. The greatest APC was observed for younger female AYAs, particularly those aged 20–24 years, APC = 10.1 (3.3–17.5), *p* = 0.014, and 25–29 years, APC = 4.9 (1.8–14.3), *p* = 0.050.

### Colonoscopy History

Colonoscopies were recorded for 77.8% (376/483) of the AYA CRC cohort, with 1,377 total hospital admissions for colonoscopy between 1982 and 2007. Almost a quarter of the cohort had no recorded colonoscopy over the study period (22.2%, 107/483). For these individuals, CRC was diagnosed during surgical procedure with no follow-up colonoscopies recorded over the study period.

**Figure 2:**
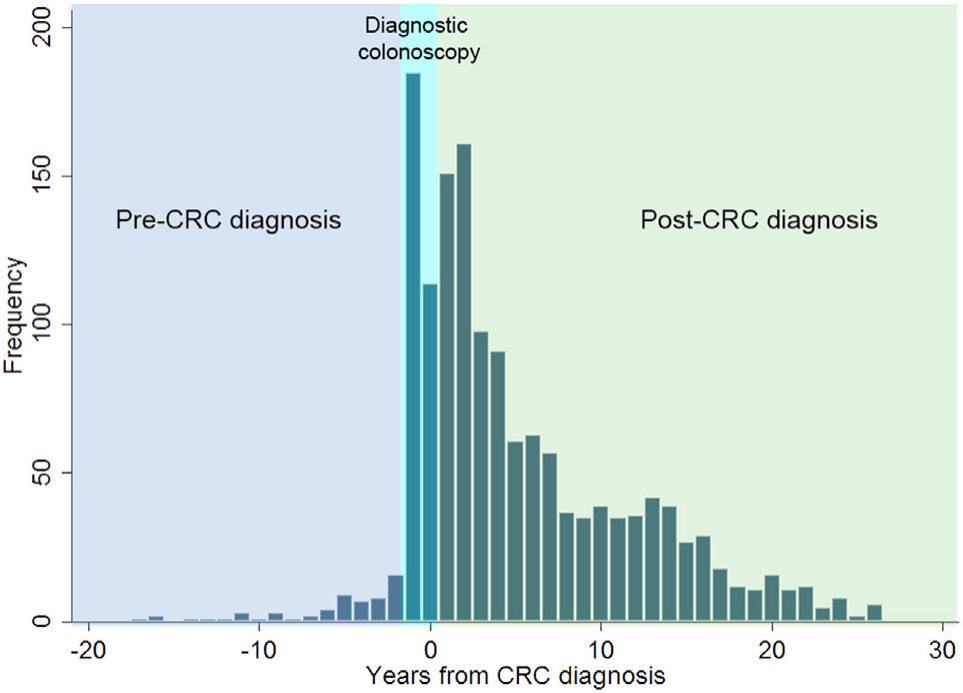
Distribution of all colonoscopies performed relative to incident colorectal cancer (CRC) diagnosis in adolescents and young adults in Western Australia during 1982–2007.

The majority of colonoscopies (70.5%, 971/1,377) were performed post-CRC diagnosis for surveillance purposes to prevent metachronous cancer (Figure 2). Colonoscopy was uncommon among AYAs prior to CRC diagnosis, with only 6.8% (33/483) of the cohort with any record of pre-diagnosis colonoscopy. Mean age at index colonoscopy for the cohort was 34.3 ± 5.7 years (range: 16–52 years). For the majority of AYAs, the index colonoscopy was performed during the hospital admission where CRC diagnosis was made (70.5%, 265/376). Only 8.8% of AYAs (33/376) had their index colonoscopy in the pre-diagnosis period, while 20.7% (78/376) had their index colonoscopy during treatment follow-up.

### Age and Tumor Grade at Diagnosis

Adolescents and young adults with a recorded pre-diagnosis colonoscopy were significantly younger at index colonoscopy (29.7 ± 6.8 years) compared to those with index colonoscopy at CRC diagnosis (34.8 ± 5.4 years), *p* < 0.001 (Table 2). AYAs with pre-diagnosis colonoscopy were also significantly older at time of incident CRC diagnosis (36.7 ± 0.7 years) compared to those with no pre-diagnosis colonoscopy (32.6 ± 0.2 years), *p* < 0.001. At CRC diagnosis, a significantly greater proportion of AYAs with pre-diagnosis colonoscopy had low grade (well-differentiated) tumors (21.2%) compared to those with no pre-diagnosis colonoscopy (5.6%), *p* = 0.001. A greater proportion of AYAs with no pre-diagnosis colonoscopy had high grade (poorly differentiated) tumors (34.1%) compared to AYAs with pre-diagnosis colonoscopy (24.2%), *p* = 0.001.

**Table 2:** Comparison of age and tumor grade at colorectal cancer (CRC) diagnosis between adolescents and young adults (AYAs) with and without pre-diagnosis colonoscopy (*n* = 483).

	Pre-diagnosis colonoscopy (*n* = 33)	No pre-diagnosis colonoscopy (*n* = 450)
**Age at index colonoscopy, *n* (%)**		
15–19 years	<5 (3.0)	0 (0)
20–24 years	<5 (9.1)	15 (4.2)
25–29 years	5 (15.2)	34 (9.5)
30–34 years	6 (18.2)	74 (20.7)
35–39 years	18 (54.6)	235 (65.6)
Mean age at index colonoscopy ± SD	33.7 ± 1.5*	37.1 ± 0.4
**Age at incident CRC diagnosis, *n* (%)**		
15–19 years	0 (0)	10 (2.1)
20–24 years	0 (0)	36 (7.4)
25–29 years	<5 (12.1)	64 (13.2)
30–34 years	10 (30.3)	133 (27.5)
35–39 years	19 (57.6)*	241 (49.8)
Mean age at incident CRC diagnosis ± SD	36.7 ± 0.7*	32.6 ± 0.2
**Tumor grade, *n* (%)**		
1: Low or well-differentiated	24 (16.2)*	62 (6.6)
2: Intermediate or moderately differentiated	9 (27.3)	142 (29.3)
3: High or poorly differentiated	8 (24.2)*	165 (34.1)
9: Not determined	9 (27.3)	149 (30.8)

*p < 0.05, significantly different from AYAs with no pre-diagnosis colonoscopy.

### Five- and Ten-Year Mortality

A total of 146 and 117 AYAs died within 5 and 10 years of incident CRC diagnosis, respectively (Table 3). There was no significant difference in all-cause 5- or 10-year mortality rates for AYAs with and without a pre-diagnosis colonoscopy. CRC-related 5-year mortality was 56% lower in the group with pre-diagnosis colonoscopy than those without, MRR = 0.44 (95% CI 0.27–0.75), *p* = 0.045. Similarly, CRC-related 10-year mortality was 57% lower for those with pre-diagnosis colonoscopy compared to those without, MRR = 0.43 (95% CI 0.24–0.83), *p* = 0.043.

**Table 3:** Five- and ten-year colorectal cancer (CRC)-related mortality for adolescents and young adults with and without pre-diagnosis colonoscopy.

			Five-year mortality (*n* = 351)			Ten-year mortality (*n* = 234)		
			
	*n*	Deaths	MRR (95% CI)	*p*	*n*	Deaths	MRR (95% CI)	*p*
**All-cause deaths**								
Pre-diagnosis colonoscopy	21	11	0.63 (0.27–1.28)	0.061	15	10	0.68 (0.09–1.46)	0.085
No pre-diagnosis colonoscopy	330	135			219	107		
**CRC-related deaths**								
Pre-diagnosis colonoscopy	21	9	0.44 (0.27–0.75)	0.045*	15	8	0.43 (0.24–0.83)	0.043*
No pre-diagnosis colonoscopy	330	131			219	100		

*p < 0.05.

*MRR, mortality rate ratio based on negative binomial regression adjusted for sex and age at incident CRC diagnosis and Charlson comorbidity index; CI, confidence interval*.

## Discussion

While the overall age-standardized incidence of CRC among AYAs in WA remains low (4.8 per 100,000) relative to the overall incidence in all age groups [62 per 100,000 in 2012 (27)], our results show a clear and significant upward trend in CRC incidence in this younger age group. Between 1982 and 2007, a 3.0% annual increase in CRC incidence was observed among AYAs in WA. In particular, CRC incidence in female AYAs rose significantly in all age groups with the exception of those aged 15–19 years. Increasing trends in CRC incidence were also observed for male AYAs, although trends were not statistically significant.

Our results are consistent with a growing number of studies demonstrating a significant increase in CRC incidence in those aged under 50 internationally. In the US, Bailey et al. (28) recently showed that at the present rate, the incidence of CRC among young adults will almost double by 2030 while simultaneously declining by more than 30% in adults over 50 years of age. The reasons underlying the rise in CRC in the younger population are currently not well understood (15, 18). However, modern Westernized lifestyle and behaviors have been implicated as potential contributors, including high consumption of takeaway and processed food and red meat in addition to obesity and low physical activity, which are known risk factors for CRC (29–31) and prevalent in contemporary Australian society (29, 32). Although smoking rates among Australian AYAs have reduced drastically over the past two decades (33), excessive alcohol consumption among AYAs has substantially increased (34) and may also partially account for the rising incidence of CRC in this population (35, 36).

Pre-diagnosis colonoscopy was uncommon among AYAs in our cohort with only 6.8% with a recorded pre-diagnosis colonoscopy and 71% being diagnosed with CRC at index colonoscopy. In Australia, national guidelines recommending routine CRC screening in adults over 50 years were introduced in 1999 with the NBCSP subsequently launched in 2006 (37). An Australian report on adults aged 45 years and above showed that screening colonoscopy was associated with a 50% reduction in risk of subsequent CRC diagnosis compared to no screening (38). In the US, successful implementation of CRC screening programs in the older population have been credited as the main driver of declining CRC rates in those aged above 50 years (5, 39). Austin et al. (5) demonstrated a significant inverse correlation between state-level APC of CRC incidence and colonoscopy rates in the US between 1998 and 2009 in adults aged 50 years and over. Specifically, states with greater reduction in CRC incidence rates over the study period tended to have higher rates of screening colonoscopy. A significant inverse correlation between CRC mortality rates and CRC screening rates between 1990–1994 and 2003–2007 has also been demonstrated in the older US population (39).

Interestingly, a number of studies have found that AYAs with CRC exhibit more advanced disease at diagnosis compared to older adults and receive more aggressive cancer treatment (15, 40–42). While it is currently unclear why this phenomenon occurs, some researchers have suggested that young-onset CRC may represent a different, more aggressive underlying disease process compared to later-onset CRC (43), although robust evidence of a more rapid adenoma-carcinoma sequence in younger adults is yet to be established. Others have implicated the absence of routine screening in this age group. As younger persons are currently omitted from routine CRC screening, CRC is typically detected in younger patients only when it becomes symptomatic or emergent and generally at more advanced stage of disease (15, 18, 20, 42). Thus, more aggressive treatment is required due to delayed diagnosis (42). Consistent with this hypothesis, we found that just under a quarter of our AYA cohort were likely emergency presentations with no admission for colonoscopy prior to CRC diagnosis and incident diagnosis made during a surgical procedure. Over 60% of our cohort had moderately or poorly differentiated tumors at CRC diagnosis. The opportunity for cancer prevention through detection and removal of premalignant lesions is also not available to young Australians. A recent study forecasted that due to late detection and accelerated progression of disease, CRC patients younger than 50 years will have the worst outcomes of any age group (20).

While colonoscopy prior to CRC diagnosis was uncommon among AYAs in our cohort, our results highlight some potential benefits of pre-diagnosis colonoscopy for younger adults, which may warrant further investigation. On average, AYAs with a pre-diagnosis colonoscopy were diagnosed with CRC at an older age relative to those with no pre-diagnosis colonoscopy history. Over 20% of AYAs with pre-diagnosis colonoscopy had well-differentiated tumors at presentation compared to only 5% of those without. Moreover, both 5- and 10-year CRC-related mortality rates were reduced by over 50% for AYAs with pre-diagnosis colonoscopy compared to AYAs without any colonoscopy history prior to diagnosis. These findings likely highlight the opportunity for early detection and removal of any premalignant adenoma through pre-diagnosis colonoscopy which could both delay CRC onset and enhance survival.

Our current findings add to an emerging body of research calling for action to address the rising incidence of CRC in the younger population (17, 18, 42). While the simplest suggestion may be to initiate average-risk CRC screening at an earlier age given the demonstrated benefits of screening colonoscopy in the older population (44), the costs and risks of widespread application of colonoscopy screening need to be carefully balanced with potential benefits (18, 44). CRC screening in average-risk persons younger than 50 years is unlikely to be cost-effective given that young-onset CRC comprises less than 7% of all CRC cases (19).

Risk-stratified screening for CRC in the average-risk population is a growing area of interest and may offer the most optimal solution (45–47). Current CRC screening models assume equal risk of CRC in the average-risk population with undifferentiated screening approaches for adults aged 50 years and above. However, research suggests that the population presently considered at “average risk” is not homogenous in terms of CRC risk and could be further stratified into distinct risk groups with tailored screening approaches and intervals for each risk level (46, 48, 49). Tailored screening for AYAs with higher than average risk for CRC likely offers a more cost-effective method of CRC screening for this group. A number of risk stratification models for advanced neoplasia and CRC have been developed in recent years; however, most are developed for the older population and their current predictive power is suboptimal (48). To better target population level screening interventions for CRC, future risk models need to simultaneously consider the average-risk population under 50 years given the demonstrated rising incidence of CRC in this age group. The challenge for researchers and policymakers remains how to best identify persons, including AYAs, at-risk of CRC and for whom early screening would be beneficial (42).

### Limitations and Directions for Future Research

Our findings show an increasing trend in CRC incidence in WA over 25 years; however, trends over the most recent decade could not be explored due to lack of post-2007 data as our analysis was based on an existing data source with end date of 2008. However, our results are consistent with other Australian and international research (11–17) showing a rising incidence of CRC in the AYA population over recent years. To date, trends in CRC incidence among Australian AYAs have only been explored to 2010 (17), with very limited other research examining colonoscopy use and costs and benefits in the younger population. Future research examining CRC incidence trends and colonoscopy uptake in Australian AYAs over the most recent decade will provide valuable insight into whether extending average risk screening into the younger population is warranted. Other limitations include we were unable to quantify the number of Lynch syndrome cases and investigate trends in hereditary vs. sporadic CRC cases over time as the WACR does not document this data, and we were unable to examine cancer stage at presentation in our analyses as this data is not collected by the WACR.

## Conclusion

In summary, our study found a growing increase in CRC incidence in AYAs in WA. Pre-diagnosis colonoscopy was rare in AYAs but where performed it was associated with later age and lower tumor grade at diagnosis and a greater than 50% reduction in CRC-related mortality within 10 years of incident diagnosis. Future research identifying strategies for early CRC detection in the AYA population is warranted.

## Ethics Statement

Ethics approval for this study was obtained from the University of Western Australia Research Ethics Committees (reference number: RA/4/1/2228).

## Author Contributions

LT designed the study, designed and performed the statistical analysis, and drafted and revised the manuscript. NS-B, AM, EM, and IL-V revised the draft manuscript. HE and PO obtained funding and revised the draft manuscript. DP obtained funding, revised the draft manuscript, and provided study supervision.

## Conflict of Interest Statement

The authors declare that the research was conducted in the absence of any commercial or financial relationships that could be construed as a potential conflict of interest.
